# Dynamic airway collapse after hybrid repair of a Kommerell diverticulum in a right-sided aortic arch: Limits of endovascular decompression

**DOI:** 10.1016/j.xjtc.2026.102385

**Published:** 2026-04-09

**Authors:** Mauin Uddin, Marco Lizwan, Muhammad Usman Shah, Azamal Ibrahim G. Mulla, Sivaraj Pillai Govindasamy

**Affiliations:** a Department of Cardiothoracic Surgery, Liverpool Heart and Chest Hospital, Liverpool, United Kingdom; b Department of Cardiothoracic Surgery, National Heart Centre Singapore, Singapore, Singapore

**Figure:**
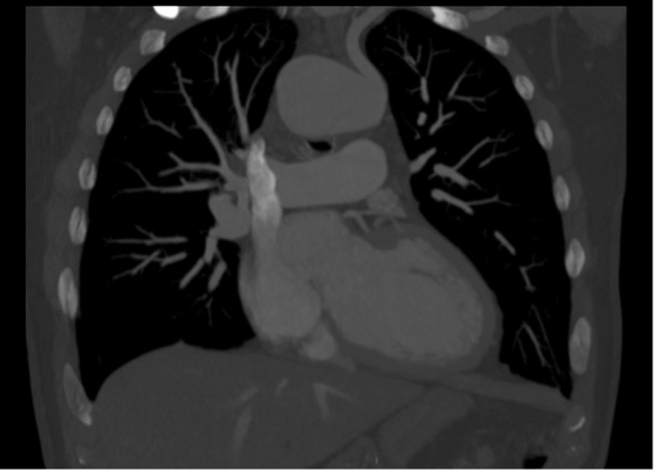
CT imaging demonstrating the anatomical relationship between the diverticulum and airway.

**Central Message**

Hybrid repair of Kommerell diverticulum may inadequately decompress dynamic airway obstruction. Early open intervention may be required.

Kommerell diverticulum (KD) represents a saccular dilatation at the origin of an aberrant subclavian artery and is commonly associated with a right-sided aortic arch (RAA) and aberrant left subclavian artery (LSCA). This rare congenital anomaly can compress adjacent mediastinal structures, including the trachea and esophagus.^1^, ^2^, ^3^ Because of its rarity and heterogenous presentation, optimal management strategies remain debated.

Management strategies remain heterogenous and include open arch reconstruction, frozen elephant trunk (FET) techniques, hybrid strategies incorporating endovascular exclusion, and staged procedures tailored to individual anatomy.^1^, ^2^, ^3^, ^4^, ^5^ Most published series focus on aneurysmal exclusion and aortic outcomes, whereas the implications for airway dynamics are less well described. We report a case of acute postoperative airway collapse after staged hybrid repair of KD, highlighting the limitations of endovascular decompression and the importance of early recognition of persistent airway compression.

## Case Presentation

Written informed consent was obtained from patient. Institutional review board approval was not required.

A 54-year-old man presented with a history of chronic cough lasting 3 to 4 months without documented infection. He denied dysphagia or stridor, and pulmonary function test findings were satisfactory. Initial radiography of the chest demonstrated a widened mediastinum, prompting further imaging.

**Figure 1:**
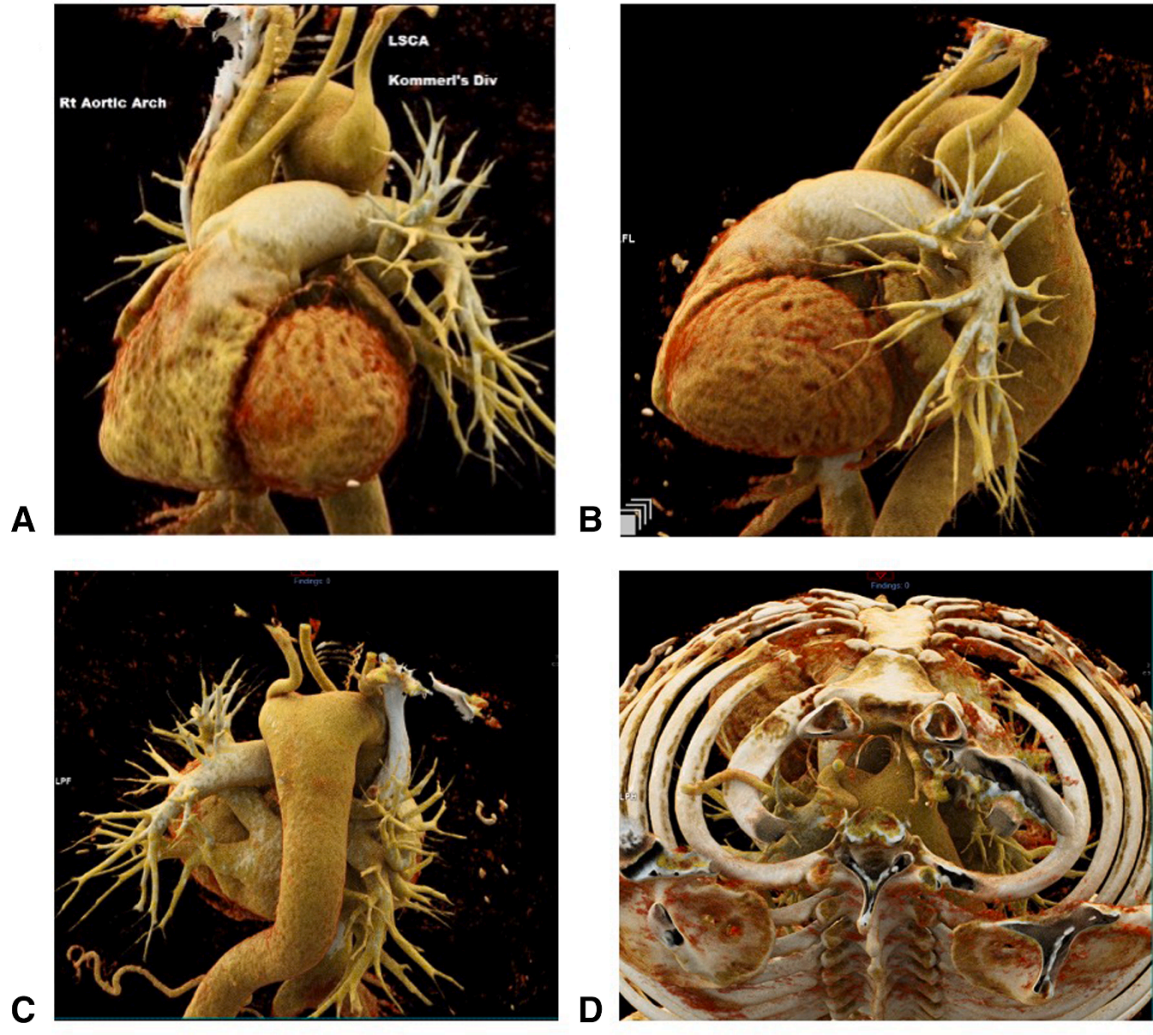
(A-D) Preoperative anatomy and hybrid intervention. Three-dimensional reconstruction demonstrating a large Kommerell diverticulum (*KD*) arising from an aberrant left subclavian artery (*LSCA*) in a right-sided aortic arch, causing posterior displacement of the trachea and esophagus.

Computed tomography (CT) revealed an RAA with a large KD arising from an aberrant LSCA, causing posterior displacement of the trachea and esophagus (Figure 1, Video 1). The recurrent respiratory infections were considered likely related to chronic airway compression and impaired airway clearance caused by the diverticulum. Echocardiography revealed severe mitral regurgitation attributable to P2 prolapse and severe aortic regurgitation.

**Figure 2:**
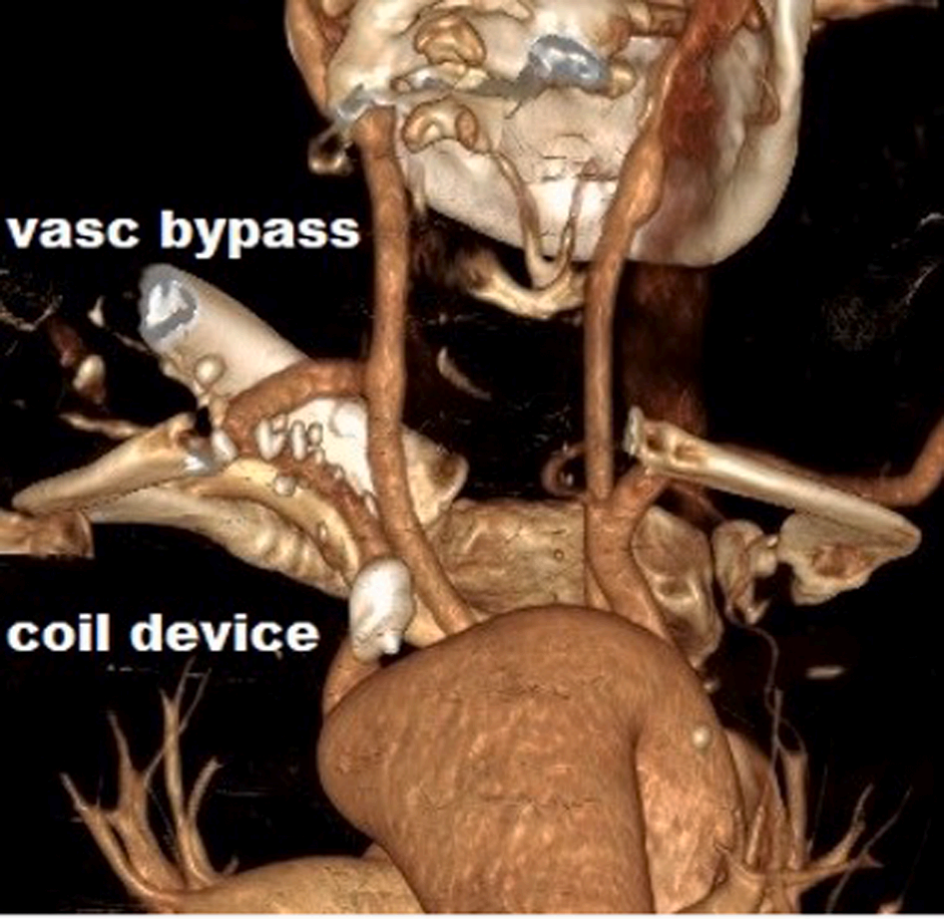
Intraoperative schematic showing left carotid–left subclavian bypass with embolization of the proximal subclavian artery to exclude inflow to the diverticulum.

After a multidisciplinary discussion, a staged surgical approach was undertaken. The first stage consisted of left carotid–left subclavian bypass using an 8-mm Dacron graft with embolization of the proximal LSCA to exclude inflow into the diverticulum (Figure 2).

One week later, the patient underwent mitral valve repair, bioprosthetic aortic valve replacement, and zone 1 aortic arch replacement using FET. Zone 1 deployment was selected to facilitate arch reconstruction while avoiding extensive dissection around the compressed airway and to allow adequate proximal landing for the stent graft while maintaining cerebral perfusion through supra-aortic debranching.

**Figure 3:**
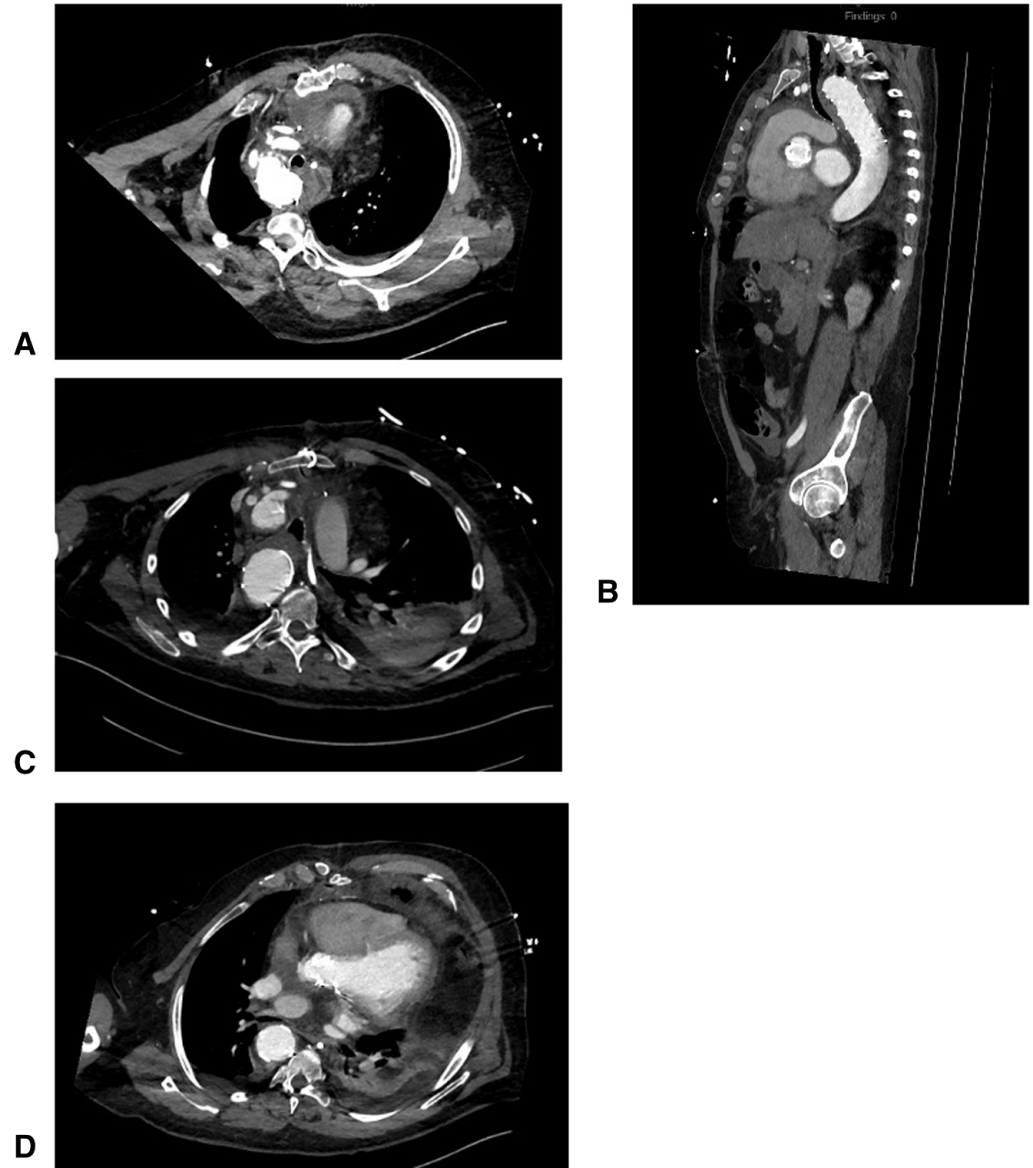
Post-frozen elephant trunk computed tomography demonstrating persistent airway compression. Axial (A, C-D) and sagittal (B) computed tomography showing a thrombosed Kommerell diverticulum located between the frozen elephant trunk and the occluded left subclavian artery with absence of endoleak. There was persistent severe compression of the distal trachea and right main bronchus.

Postoperatively, the patient was extubated but rapidly developed severe inspiratory stridor and respiratory distress requiring urgent reintubation. Urgent CT and bronchoscopy revealed marked distal tracheal collapse with complete occlusion of the right main bronchus (Figure 3). Initial LSCA embolization demonstrated brisk retrograde flow into the diverticulum on angiography, necessitating additional coil deployment to achieve complete occlusion. Subsequent CT imaging confirmed absence of endoleak. However, despite successful exclusion, airway compression persisted, indicating that residual mass effect rather than ongoing pressurization was the dominant mechanism contributing to the collapse.

**Figure 4:**
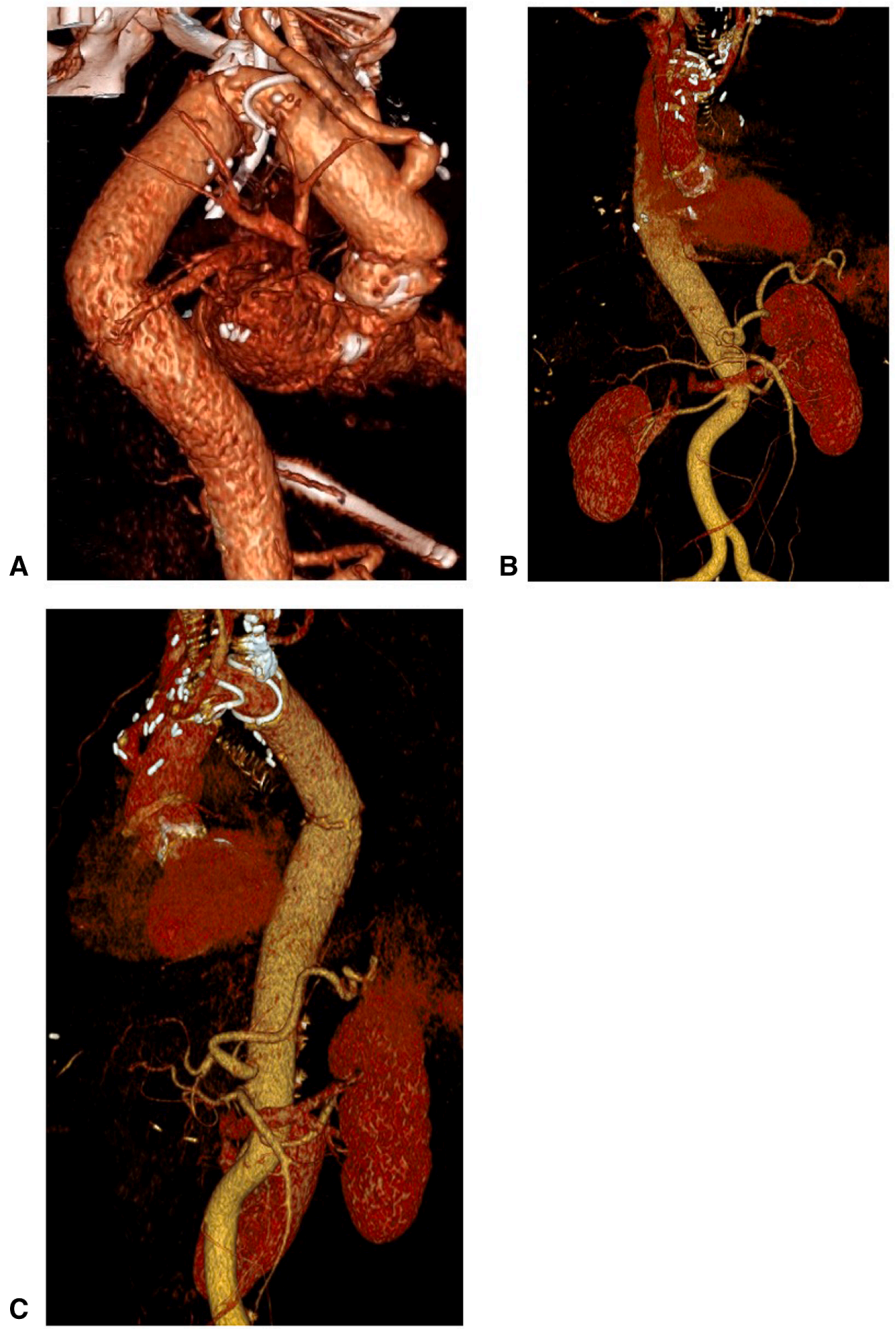
(A-C) Postoperative reconstruction. Completion 3-dimensional reconstruction demonstrating the aortic arch and descending thoracic aorta after frozen elephant trunk and distal arch replacement, with relief of airway compression and restored vascular continuity.

Urgent open repair was therefore undertaken via right posterolateral thoracotomy through the fifth intercostal space. The distal arch and proximal descending thoracic aorta were replaced, and the FET was truncated proximal to the LSCA origin to eliminate residual mass effect. Postoperative bronchoscopy demonstrated complete resolution of airway obstruction. The patient was extubated uneventfully. Completion imaging confirmed decompression of the tracheobronchial tree and satisfactory aortic reconstruction (Figure 4). Bronchoscopic images were not preserved and therefore could not be included here.

## Discussion

This case highlights a critical limitation of hybrid repair strategies for KD in the presence of airway compression. Although total arch replacement with FET and staged hybrid approaches have been reported with favorable outcomes, most reports emphasize on aortic reconstruction rather than airway dynamics.^1^, ^2^, ^3^, ^4^, ^5^ In our patient, significant radiologic airway compression was present preoperatively but was clinically compensated.

Serial postoperative CT imaging demonstrated persistent airway compression despite complete thrombosis of the diverticulum and absence of endoleak. These findings suggest that airway compromise was not driven by ongoing pressurization but rather by static mass effect from the thrombosed diverticulum, distal arch, and proximal descending thoracic aorta. In addition, thrombus formation between the FET and aortic wall may have contributed to a transient increase in rigidity and external compressive forces. Importantly, exclusion of the LSCA and diverticulum did not relieve airway compression, suggesting that decompression strategies targeting the diverticulum alone may be insufficient when multiple anatomic structures contribute to airway compromise.

Preoperative imaging features that may increase the risk of postoperative airway compromise include severe distal tracheal or bronchial compression, right bronchus displacement, large diverticulum diameter, and arch configurations in which the distal arch or proximal descending thoracic aorta contributes to airway encirclement. In such patients, hybrid repair alone may not provide adequate decompression.

Although airway compression from KD has been described previously, reports of new or worsening airway collapse after hybrid or endovascular repair remain rare. Our case highlights that exclusion of diverticulum alone may not adequately address airway pathology.

Bronchoscopy proved essential for diagnosis and guided timely escalation to definitive open repair. Early bronchoscopy should be considered when unexplained respiratory deterioration occurs after hybrid arch procedures.

## Conclusions

KD associated with an RAA may cause dynamic airway compression. Hybrid exclusion of the diverticulum may not reliably decompress the airway when distal arch or descending aortic structures contribute to compression. Early bronchoscopy and a low threshold for definitive open distal arch and proximal descending thoracic aorta replacement are critical when airway compromise persists.

## Conflict of Interest Statement

The authors reported no conflicts of interest.

The *Journal* policy requires editors and reviewers to disclose conflicts of interest and to decline handling or reviewing manuscripts for which they may have a conflict of interest. The editors and reviewers of this article have no conflicts of interest.

## Supplementary Data

Video 1 Preoperative computed tomography. Video available at: https://www.jtcvs.org/article/S2666-2507(26)00192-6/fulltext.
